# Editorial: The synaptic basis of neuropathology

**DOI:** 10.3389/fnsyn.2022.1043480

**Published:** 2022-10-13

**Authors:** Fereshteh S. Nugent, Alfredo Kirkwood, Carl R. Lupica, P. Jesper Sjöström

**Affiliations:** ^1^F. Edward Hébert School of Medicine, Uniformed Services University, Bethesda, MD, United States; ^2^Department of Neuroscience, Mind/Brain Institute, Johns Hopkins University, Baltimore, MD, United States; ^3^National Institute on Drug Abuse Intramural Research Program, National Institutes of Health, Baltimore, MD, United States; ^4^Brain Repair and Integrative Neuroscience Program, Department of Medicine, Department of Neurology and Neurosurgery, Centre for Research in Neuroscience, The Research Institute of the McGill University Health Centre, Montreal General Hospital, Montreal, QC, Canada

**Keywords:** synapse, plasticity, electrophysiology, microscopy, synaptic dysfunction, GABAergic, glutamatergic

## Introduction

A growing body of evidence points to malfunctioning synapses and plasticity as key contributors to neuropathology, leading to the idea that there may be a synaptic basis for brain disease (Lüscher and Isaac, 2009; Kilgard, 2012). For example in epilepsy, recurring excessive neuronal activity in the healthy brain can recruit plasticity and result in a pathologically increased susceptibility for seizures (Cela and Sjöström). Both presynaptic (Van Battum et al., 2015; Perrone-Capano et al., 2021) and postsynaptic compartments (Kasai et al., 2021) have been implicated in neuropathology, including autism, amyotrophic lateral sclerosis, Alzheimer's disease, and schizophrenia. Since different synapse types have distinct forms of short and long-term plasticity (Blackman et al.; Larsen and Sjöström, 2015), approaching major brain disease as synaptic neuropathology may increase the likelihood of finding new and more specific treatments. For example, blocking calcium-permeable but not calcium-impermeable AMPA receptors of the cochlea can protect from excitotoxicity due to acoustic overexposure without impairing hearing (Walia et al.).

Here, we organized a Research Topic to highlight the latest advancements in the field of synaptic disease. These articles describe the state of the art by outlining recent developments and major accomplishments.

## Papers in this collection

### Synapses, plasticity, and learning

Although it is widely accepted that AMPA receptor trafficking is key for expression of long-term potentiation (LTP) at Schaffer collateral synapses to CA1 pyramidal neurons (Díaz-Alonso and Nicoll, 2021), the involvement of specific AMPA receptor domains remains unclear. To address the requirement of GluA1 AMPA receptor subunit in the developmental expression of LTP, Liu et al. used their newly generated knock-in mouse line (GluA1^C2KI^; Zhou et al., 2018), where the C-terminal domain (CTD) of endogenous GluA1 is replaced by that of GluA2. Unlike the global or conditional genetic deletion of GluA1, the expression of the GluA1 subunit still occurs in these mice, which limits the aberrant formation of AMPA receptor complexes. Using wildtype and GluA1^C2KI^ mouse slices, they showed that the CTD of GluA1 contributed differentially to different forms of LTP.

The Kv family of voltage-gated potassium channels (VGKC) plays an important role in spike generation and propagation in the brain. Several heterozygous mutations in KCNA2, the gene encoding KV1.2 VGKC, have been identified in patients with severe epilepsy (Syrbe et al., 2015; Masnada et al., 2017; Nilsson et al., 2022). For example, in VGKC antibody-associated encephalopathy, high titers of patient-derived autoantibodies against VGKC complex may increase pathogenicity (Vincent et al., 2004). In this collection, Kirschstein et al. investigated in rats the pathogenic effects of patient-derived antisera against Kv1.2 and the VGKC-associated protein contactin-associated protein-2 (CASPR2) by stereotactic antisera microinjection into rat hippocampus. At Schaffer collateral-CA1 synapses, but not medial perforant path-dentate gyrus connections, injection of anti-Kv1.2 but not anti-CASPR2 increased neurotransmitter release, which facilitated post-synaptic depolarization and boosted LTP in CA1 but not the dentate gyrus. Moreover, both anti-Kv1.2 and anti-CASPR2 sera promoted epileptic discharges and hyperexcitability in hippocampus. The authors conclude that although the cellular effects of patient-derived Kv1.2 and CASPR2 antibodies may differ, both presented pathophysiologically relevant VGKC complex-antibodies.

Emerging studies report that fear acquisition alters excitability of infralimbic cortex neurons (Soler-Cedeno et al., 2016; Bloodgood et al., 2018), even though auditory fear conditioning does not elicit infralimbic cortex synaptic plasticity (Pattwell et al., 2012; Sepulveda-Orengo et al., 2013). Rather, plasticity in infralimbic cortex occurs after extinction but not acquisition of fear learning (Pattwell et al., 2012; Sepulveda-Orengo et al., 2013). Still, in male rats, there is evidence for reduced NMDA receptor currents at connections from ventral hippocampus to infralimbic cortex after fear learning (Soler-Cedeno et al., 2016). Castillo-Ocampo et al. wondered if females showed similar plasticity, and therefore tagged these synapses with enhanced yellow fluorescent protein, EYFP. To assess NMDA receptor levels, the obligatory subunit GluN1 was labeled. Using fluorescence-activated cell sorting of infralimbic cortex synaptosomes, the authors surprisingly showed more EYFP+/GluN1+ synaptosomes with greater average expression of GluN1 in male rats after auditory fear conditioning, but not after contextual fear conditioning, indicating a plasticity that was specific to learning paradigm. However, no such NMDA receptor plasticity was found in female rats, suggesting sex-specific auditory fear conditioning mechanisms. The increasing evidence for sex-specific differences in fear learning highlights a need for reassessment since most studies to date have disregarded this possibility.

### Neurodevelopment

Taurine is a free amino acid highly abundant in the CNS, particularly early in neurodevelopment, when it has been considered to be involved in trophic functions. Using cell cultures from vertebrate (rat) and invertebrate (*Lymnaea*) models, Mersman et al. examined how taurine affects neurite outgrowth, synapse formation, and synaptic transmission. In rat cell culture, immunocytochemistry revealed that taurine promotes neurite outgrowth and expression of synaptic markers. In *Lymnaea* cell cultures, electrophysiology revealed that taurine promotes formation of new functional synapses. These findings suggest the involvement of taurine in synaptic development mechanisms that are common across phyla.

The brain stem structure known as the lateral dorsal tegmental nucleus (LDT) has long been recognized for its central role in the brain's reticular activating system, where it may mediate sleep, wakefulness, and arousal (Jouvet, 1965). Later studies established that LDT cholinergic neurons, rather than glutamatergic or GABAergic cells, are especially important in this regard (Baghdoyan et al., 1987; Grant and Highfield, 1991; Kayama et al., 1992; Van Dort et al., 2015). In this respect, the LDT was envisaged to be important in regulating homeostatic autonomous brain functions, and resistant to experience-dependent plasticity. However, here Kohlmeier and Polli propose that more recent evidence supports the idea that the LDT and its associated synaptic pathways can undergo significant remodeling as a result of experience and exposure to abused drugs such as nicotine and cannabis. Thus, the authors identify altered synaptic plasticity, neuronal activity, and connectivity of the LDT resulting from prenatal or postnatal stimuli, and they argue that this may alter the LDT's impact on behavior.

Clmp is a synaptic adhesion molecule whose expression peaks neonatally. In the CNS, Clmp also associates with subunits of AMPA receptors and kainate receptors. Jang et al. analyzed the synaptic and behavioral effects of genetically ablating Clmp in synaptic function. In hippocampal CA3 neurons, Clmp deletion increased the frequency and amplitude of miniature excitatory post-synaptic currents (mEPSCs) mediated by AMPA receptors and kainate receptors. Behaviorally, the deletion enhanced novel object recognition as well as susceptibility to kainate-induced seizures. These findings reveal that Clmp negatively regulates fast synaptic transmission with behavioral consequences.

### Autism spectrum disorder

In autism spectrum disorders (ASD), learning of social behaviors and development of social skills are severely compromised (Chevallier et al., 2012). ASD-linked mutations in multiple genes converge on common molecular and cellular pathways that lead to synaptic and neural circuit dysfunction. A major signaling pathway implicated in ASD is β-catenin (Caracci et al.). In this collection, Alexander et al. generated mouse lines with either β-catenin up- or down-dysregulation in cortical glutamatergic neurons to provide direct behavioral evidence for a causal role of β-catenin dysregulation in ASD. To link β-catenin dysregulation in forebrain excitatory neurons to behavioral deficits, they utilized CamKIIα Cre driver mice during an early postnatal period critical for synapse formation and connectivity of neural circuits relevant to ASD. They found that β-catenin upregulation led to social deficits, increased repetitive behaviors, and aberrant expression of ADS-linked genes, whereas β-catenin downregulation prevented behavioral and molecular ASD phenotypes. The study suggests that the β-catenin pathway represents a point of convergence for ASD-linked genes that contribute to synaptic and circuit dysfunction and social deficits.

Cytoplasmic FMR1-interacting protein 1 (*Cyfip1*) and Synaptic Ras GTPase-activating protein 1 (*SynGAP1*) are both important for brain development and function, and there are similarities of action between *Cyfip1* and *SynGAP1* suggesting that haploinsufficiency of either causes intellectual disability or autism (Chen et al., 1998; Kushima et al., 2018). Consequently, Sahasrabudhe et al. explored whether *Cyfip1* haploinsufficiency impacted *SynGAP1* levels and localization. The authors found that in mouse hippocampus, Cyfip1 indeed regulated SynGAP1 anchoring and synaptic localization, and that the two proteins together direct actin polymerization and GAP activity at synapses. Compared to wildtype mice, *Cyfip*1^+/−^ mice had reduced levels of SynGAP1 and GluA1 at synapses, whereas mGluR1/5, GluA2, and F-actin were enhanced. These findings nicely demonstrate how graded changes in levels of a specific protein can effectively be boosted locally at synapses, resulting in an overall large impact.

Banke and Barria investigated the early postnatal development of synaptic function and plasticity in Fmr1 knock-out (KO) mouse, a model of Fragile X syndrome (FXS), the leading monogenic form of ASD. Their electrophysiological measures revealed two prominent features: a large but transient enhanced GluA2 subunit expression of synaptic AMPA receptors that it is over by the 2nd postnatal week, and the virtually complete absence of LTP in the first 3 weeks of age. The results are discussed in the context of the known abnormalities in spine density and shape of Fmr1 KO mice that already manifest at that developmental stage.

### Injury and inflammation

The neuropeptide corticotropin releasing factor (CRF, also known as CRH) is widely known for its stress-induced activation of the hypothalamic-pituitary-adrenal axis, but CRF also acts in the brain to directly regulate both positive and negative motivated behavioral response to stressors (Lemos et al., 2012; Lemos and Alvarez, 2020; Baumgartner et al., 2021). Persistent CRF dysregulation following mild traumatic brain injury (mTBI) has been suggested to contribute to chronic psychiatric morbidities with abnormal stress-related neuronal responses and negative affective states associated with mTBI (Fox et al., 2016; Russell et al., 2018; Kosari-Nasab et al., 2019). However, it is unclear how mTBI alters synaptic and neuronal function of CRF neurons. In this collection, Simmons et al. investigated cell-type and sub-region-specific effects of mild blast traumatic brain injury (mbTBI) on CRF neuronal and synaptic function in ventral and dorsal hypothalamic paraventricular nucleus (vPVN and dPVN) using whole-cell recordings in adult male mice a week after injury. While mbTBI did not affect neuronal excitability of CRF neurons in dPVN and vPVN sub-regions, it selectively increased spontaneous firing of dPVN CRF neurons due to mbTBI-induced GABAergic dysfunction. The authors suggest that mbTBI-induced dysregulation of non-neuroendocrine CRF pathways from the dPVN underlies anxiety-like behaviors associated with this model of mTBI (Russell et al., 2018). Their work also highlights that GABAergic synaptic inputs may be more susceptible to mTBI resulting in dysfunction of distinct central CRF neural circuits implicated in mood disorders following mTBI.

Although hot compresses are often used to treat pain and inflammation associated with peripheral nerve damage, the mechanism of this beneficial treatment is largely unknown. In a study by Chan et al. in this collection, behavioral measures and a series of molecular markers of nerve damage were obtained in rats undergoing sciatic nerve chronic constriction injury (CCI) that received either hot compress treatment or no intervention. The investigators found that 40°–42° C compresses reduced inflammation and restored sciatic nerve sensitivity, and that an increase in hyperalgesia seen after this nerve injury was also reduced along with synaptophysin, a protein that may mediate sciatic nerve pain through activation of spinal dorsal horn neurons. Hot compress treatment also dramatically reduced several proinflammatory cytokines, both at the site of injury and in the brain. Therefore, this study supports the use of hot compresses to treat sciatic nerve pain.

### Neurodegenerative disease

Contemporary research in psychiatry has re-ignited interest in the use of psychedelics to treat disorders ranging from depression to anxiety. Psychedelic drugs are agonists at serotonin type 2A receptors (5-HT2A), which are found in high concentrations in brain areas associated with attention, introspection, and memory (e.g., cerebral cortex, hippocampus, and amygdala). Here, Vann Jones and O'Kelly review the use of psychedelics in the treatment of psychiatric disorders and evaluate the hypothesis that they may also provide therapy for dementia and Alzheimer's disease. The authors discuss evidence showing that psychedelics can increase brain metabolism, decrease brain inflammation, and enhance cognitive performance in healthy human subjects, supporting this potential therapy for Alzheimer's patients.

In humans, Alzheimer's disease has a long presymptomatic phase. In contrast, most mouse models—like those based on Aβ precursor protein (APP) transgene to drive pathology *via* oligomeric amyloid-β (Aβ)—often overexpress the molecule of interest, thus greatly accelerating the process. To overcome this limitation, Hrynchak et al. studied the progression of pathology in transgenic mice expressing equal levels of mouse and human APP carrying (FAD)-linked mutations, a line that develops plaques only in old age. The analysis revealed differences across brain regions. Increasing concentrations of Aβ lowered spine density and changed spine morphology in hippocampal CA1 neurons but had no effect in neocortical pyramidal cells.

Synaptic degeneration occurs early in many neurodegenerative pathologies such as Huntington's and Parkinson's diseases (Cheng et al., 2010; Milnerwood and Raymond, 2010), but the mechanisms of synaptic dysfunction and loss are not well-understood. Mounting evidence has implicated Wnt deficiency in adult synaptic degeneration as well as in neurodegenerative diseases (Galli et al., 2014; Liu et al., 2014). To manipulate Wnt signaling in mouse striatum, Galli et al. induced expression of the Wnt antagonist Dickkopf-1. This led to a loss of inhibitory synapses on striatal medium spiny neurons and affected the synaptic transmission of D2 striatal medium spiny neurons. Conversely, turning off Dickkopf-1 to reactivate Wnt resulted in complete recovery of inhibitory and dopamine synapse numbers. Although Wnt signaling has not yet been directly linked to Parkinson's or Huntington's, the findings of Galli et al. highlight Wnt as a promising therapeutic target for brain repair in these neuropathologies.

With a similar rationale, Iuliano et al. performed electron microscopy and biochemical analyses of synaptic proteins and lipids in mouse models of Huntington's disease to identify several pre and postsynaptic molecules whose distribution and concentrations were altered. Their study strengthens the idea that the Huntington's mutation causes age-dependent disruption of the localization and composition of synaptic proteins and lipids that are key to synaptic function. Their findings furthermore support the notion these changes in Huntington's mice reflect those in patients that ultimately lead to cognitive and psychiatric pathology.

### Concluding remarks

By providing a thorough overview, this Research Topic sheds light on recent progress made in the synaptic disease field as well as on its future challenges. We hope this collection will inform and inspire future research on the synaptic basis of neuropathology.

## Author contributions

FN, AK, CL, and PS wrote the manuscript. All authors contributed to the article and approved the submitted version.

## Funding

FN was supported by the National Institutes of Health (NIH)—National Institute of Neurological Disorders and Stroke (NIH/NINDS) Grant#R21 NS120628. AK was supported by RO1-EY012124, PO-AG009973, and RO1-EY025922. CL was supported by DA000599 and the National Institutes of Health, National Institute on Drug Abuse Intramural Research Program. PS was supported by FRQS *Chercheurs-Boursiers* Senior 9 Award #254033.

## Conflict of interest

The authors declare that the research was conducted in the absence of any commercial of financial relationships that could be construed as a potential conflict of interest.

## Publisher's note

All claims expressed in this article are solely those of the authors and do not necessarily represent those of their affiliated organizations, or those of the publisher, the editors and the reviewers. Any product that may be evaluated in this article, or claim that may be made by its manufacturer, is not guaranteed or endorsed by the publisher.

## Author disclaimer

The opinions and assertions contained herein are the private opinions of the authors and are not to be construed as official or reflecting the views of the Uniformed Services University of the Health Sciences or the Department of Defense or the Government of the United States.
